# Results from a blind and a non-blind randomised trial run in parallel: experience from the Estonian Postmenopausal Hormone Therapy (EPHT) Trial

**DOI:** 10.1186/1471-2288-12-44

**Published:** 2012-04-04

**Authors:** Piret Veerus, Krista Fischer, Matti Hakama, Elina Hemminki

**Affiliations:** 1 Department of Epidemiology and Biostatistics, National Institute for Health Development, Hiiu 42, 11619 Tallinn, Estonia; 2 Estonian Genome Center, University of Tartu, Tiigi 61b, 50411 Tartu, Estonia; 3 Tampere School of Public Health, University of Tampere, Medisiinarinkatu 3, FIN-33520 Tampere, Finland; 4 National Institute for Health and Welfare (THL), P.O. Box 30, FIN-00271 Helsinki, Finland

**Keywords:** Clinical trial, Blinding, Internal and external validity

## Abstract

**Background:**

The Estonian Postmenopausal Hormone Therapy (EPHT) Trial assigned 4170 potential participants prior to recruitment to blind or non-blind hormone therapy (HT), with placebo or non-treatment the respective alternatives. Before having to decide on participation, women were told whether they had been randomised to the blind or non-blind trial. Eligible women who were still willing to join the trial were recruited. After recruitment participants in the non-blind trial (N = 1001) received open-label HT or no treatment, participants in the blind trial (N = 777) remained blinded until the end of the trial. The aim of this paper is to analyse the effect of blinding on internal and external validity of trial outcomes.

**Methods:**

Effect of blinding was calculated as the hazard ratio of selected chronic diseases, total mortality and all outcomes. For analysing the effect of blinding on external validity, the hazard ratios from women recruited to the placebo arm and to the non-treatment arm were compared with those not recruited; for analysing the effect of blinding on internal validity, the hazard ratios from the blind trial were compared with those from the non-blind trial.

**Results:**

The women recruited to the placebo arm had less cerebrovascular disease events (HR 0.43; 95% CI: 0.26-0.71) and all outcomes combined (HR 0.76; 95% CI: 0.63-0.91) than those who were not recruited. Among women recruited or not recruited to the non-treatment arm, no differences were observed for any of the outcomes studied.

Among women recruited to the trial, the risk for coronary heart disease events (HR 0.77; 95% CI: 0.64-0.93), cerebrovascular disease events (HR 0.66; 95%CI: 0.47-0.92), and all outcomes combined (HR 0.82; 95% CI: 0.72-0.94) was smaller among participants in the blind trial than in the non-blind trial. There was no difference between the blind and the non-blind trial for total cancer (HR 0.95; 95% CI: 0.64-1.42), bone fractures (0.93; 95% CI: 0.74-1.16), and total mortality (HR 1.03; 95% CI: 0.53-1.98).

**Conclusions:**

The results from blind and non-blind trials may differ, even if the target population is the same. Blinding may influence both internal and external validity. The effect of blinding may vary for different outcome events.

**Trial registration:**

[ISRCTN35338757]

## Background

Evidence from randomised controlled trials (RCTs) is an essential source of information used by public health policy makers as well as by clinicians and patients for making treatment decisions. RCTs are conducted against the backdrop of difficult methodological choices related to the balance between internal validity (reliability of the results) and external validity (generalisability) [1].

Internal validity is maximized by reducing bias by using randomisation and blinding [2-4]. Most trial methodology concentrates on issues related to internal validity [5]. There are no general guidelines as to how the external validity of randomised controlled trials should be assessed [6-8]. Issues that potentially affect external validity are considered to be the trial setting, the selection of patients and clinicians, differences between the trial protocol and routine practice, the relevance of outcome measures and the length and completeness of follow-up [9,10].

The Estonian Postmenopausal Hormone Therapy (EPHT) Trial was carried out from 1999 to 2004 in order to study the impact of postmenopausal hormone therapy (HT) on bone fractures, cardiovascular diseases, and cancer [11], on health services utilisation and health care costs [12], on symptom reporting and quality of life [13]. An additional aim was to study the impact of blinding on recruitment [14], adherence [15] and trial outcomes. Therefore, all potentially eligible participants were simultaneously randomised to four trial arms before signing the informed consent: blind and non-blind HT arms, placebo arm or non-treatment arm.

The earlier published results from the EPHT Trial with the follow-up until 2003 showed a difference between the blind and non-blind trial in the number of coronary heart disease events, cerebrovascular disease events and all outcomes combined among recruited women, but not for cancer and bone fractures [11]. Such observed differences in health outcomes between the blind and the non-blind trial could not be explained by the difference in adherence and contamination rates which were similar in all trial arms. This generates a hypothesis that blinding might influence validity of results from placebo-controlled, double-blind randomised trials.

In order to analyse the effect of blinding on external validity of trial outcomes, the outcomes from recruited women in the placebo and the non-treatment arms were compared with those who were not recruited; also, the numbers and the proportion of women with different background characteristics recruited to blind and non-blind trial were compared. For analysing the effect of blinding on internal validity, the outcomes from the blind trial were compared with those from the non-blind trial, and in addition separately for blind versus non-blind HT arm, and placebo versus non-treatment arm. The follow-up period was extended up to year 2007.

## Methods

### Trial population

Information about the trial and a questionnaire were mailed in 1998 to a sample of 39 713 women aged 45-64 taken from the Estonian Population Registry and living in two Estonian counties. The recruitment questionnaire included questions about willingness to join a randomised trial and questions about health and social status. Of the 14 743 women who returned the questionnaire, 6606 respondents were interested in participating, of which 4295 women were found to be eligible according to the preliminary assessment, based on time since menopause and other health data from the questionnaires.

### Randomisation

Randomisation was carried out before recruitment in permuted blocks, each of a size 16 and each block of the three trial clinics separately, at the National Research and Development Centre for Welfare and Health in Finland. The 125 women participating in the pilot study were not included in the present analysis. The 4170 eligible women who were willing to join the main trial were randomised into blind and non-blind arms and, at the same time, into treatment and control arms (two-by-two design). Hence, there were four study arms: 1) blind HT arm; 2) non-blind HT arm; 3) placebo arm and 4) non-treatment arm. Hereafter, blind HT and placebo arm combined together will be named blind trial, and non-blind HT and non-treatment arms combined together non-blind trial.

Detailed descriptions of recruitment, inclusion and exclusion criteria, trial treatment, adherence, follow-up and trial outcomes as well as the content of information leaflets and trial questionnaires have been published elsewhere [11-17]. All participants gave written informed consent. The study protocol was approved by the Committee of Medical Ethics in Tallinn, Estonia and by the Ethics Committee of the University Clinic of Tampere, Finland. Data about randomised women who were not recruited to the trial were acquired with a special permission from the Estonian Data Protection Agency.

### Recruitment, trial treatment and adherence

The 4170 randomly assigned women were mailed an invitation letter revealing whether they had been assigned to the blind or the non-blind trial. The treatment allocation was enclosed in a non-transparent sealed envelope with a woman's study number and name on it, and sent to the trial clinic. In the blind trial, the women were told that they would be using either hormone therapy or a placebo; in the non-blind trial, they were told that they would be receiving hormone therapy or non-treatment.

Final recruitment took place between January 1999 and December 2001. A total of 2323 women responded to the mailed invitation to visit the trial doctor. After this secondary assessment of eligibility, 1778 women proved to be eligible and were willing to join the trial, and their randomisation envelope was opened. The reasons for ineligibility after secondary assessment have been reported in detail elsewhere [12].

**Figure 1:**
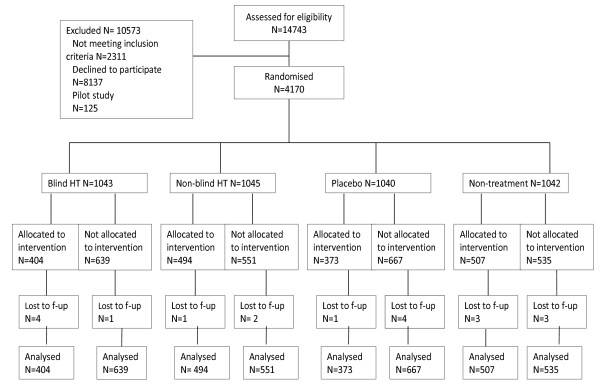
**EPHT Trial flow chart**.

As a result, 404 women were recruited into the blind HT arm, 373 into the placebo arm, 494 into the non-blind HT arm and 507 into the non-treatment arm (Figure 1). None of the trial participants switched the trial arm after randomisation. The women in the non-blind HT arm were allocated to open-label HT, the women in the non-treatment arm did not receive any drugs. Adherence was assessed by the number of collected and returned drugs and by the information from annual questionnaires and weekly reports from the clinics. Women taking more than 80% of the allocated drugs were considered to be adherent, whilst women in the non-treatment arm were considered to be adherent if they were not taking hormone therapy for 80% of the time. Data about prescribed HT use in the non-treatment and placebo arms was obtained from the Estonian Health Insurance Fund.

### Intervention

During the treatment period, participating women and health care providers were blinded to the treatment assignment in the blind trial, but not in the non-blind trial. The persons doing the linkages in registries, data collectors and data analysts at research centres were unaware of the treatment allocation, and remained blinded also after the end of the trial. After publication of the results from the Women's Health Initiative (WHI) trial, trial treatment was stopped gradually between January and May 2004. Participants in the blind trial received a letter containing information on their treatment allocation within one month of their final visit.

### Outcome measures, data acquisition and analyses performed

The randomised women were followed by annual linkages to the Estonian Health Insurance Fund database, the Estonian Cancer Registry database, and the Estonian Mortality database using a person's identity code. The outcomes studied were coronary heart disease (I20-I25 according to the 10^th ^revision of the International Classification of Diseases), [18] cancer (C00-C97), cerebrovascular disease (I60-I69), bone fractures (S12, S22, S32, S42, S52, S62, S72, S82, S92), death from all causes and all these combined. As the database of the Estonian Health Insurance Fund was considered not fully complete for the years 1999 and 2000, the first follow-up date for all outcomes in the present analysis was January 1, 2001. The results of the analysis are reported here separately for December 31, 2004 and December 31, 2007, as the last follow-up dates.

The Estonian Health Insurance Fund is the only organization in Estonia dealing with compulsory health insurance [19]. It stores information about all health care contacts, using an individual's personal identification code recorded in a centralised, computerised database. The Health Insurance Fund pays for all health care visits, diagnostic examinations, preventive and treatment procedures, hospital stays, surgeries, technical aids during or after surgery, and compensations for medicinal products. All participants in the trial were insured. As compensation to clinics depends on the transmission of data to the central electronic database and not on the diagnosis, we assume the probability of missing data in the database of the Estonian Health Insurance Registry to be minimal. The Estonian Cancer Registry database has been validated for completeness of registration, with the overall completeness of registration being 90.8% in 1998 [20].

The linkage with the Population Registry showed that during the follow-up period nine women left from Estonia (all among recruited participants: four in the blind HT arm, one in the non-blind HT arm, one in the placebo arm, three in the non-treatment arm). Data about these women in the registries might have been incomplete (Figure 1).

For analysing the effect of blinding on the selection bias (i.e. external validity), the numbers of women recruited to the blind and non-blind trial as well as the proportion of women with different background characteristics recruited to blind and non-blind trial were compared. In addition, hazard ratios for different outcome events among recruited versus non-recruited women were calculated in the placebo arm and in the non-treatment arm. The comparison was restricted to the non-HT arms and the hazard ratios between recruited and non-recruited women in the blind and non-blind HT arms were not calculated in order to exclude the effect of HT from the analysis of blinding.

For analysing the effect of blinding on reporting bias, observer bias, and placebo effect (i.e. internal validity), the hazard ratios of outcome events among recruited women in the blind and non-blind trial arms were compared, separately until the year 2004 and 2007. During the first period, participants in the HT arms were allocated to trial treatment, and the participants in trial arms as well as their physicians were blinded about their treatment allocation. Blinding and trial treatment was stopped in 2004. The analysis with the longer follow-up period until 2007 was done to check if the differences between the outcomes hold also after stopping blinding and trial treatment.

### Statistical analysis

The effect of blinding on the recruitment process was first assessed by comparing the proportions of women recruited to the blind and non-blind trial. Next, the proportion of women with different background characteristics was calculated in blind and non-blind trial arms both among recruited and non-recruited women. The baseline covariates used in the analysis (also in the adjusted analysis for the outcomes) were age, education, living place (corresponding to the clinic of recruitment), smoking status, and time since menopause.

The outcome data were analysed by time-to-event methods. For each of the target disease groups, the number of days from recruitment to the first diagnosis in this group, as registered in the Health Insurance Fund database, the Estonian Cancer Registry database, or in the Estonian Mortality database was used as the outcome variable. Time to diagnosis was censored for women who did not have the corresponding diagnosis registered during follow-up. Person-years at risk were calculated from January 1, 2001 to December 31, 2007 (and from January 1, 2001 to December 31, 2004) or to the outcome event studied or to death whichever came first.

Cumulative hazard plots based on the Kaplan-Meier method were obtained for descriptive comparison of outcome distributions in blind and non-blind trial, separately for recruited and non recruited women.

Comparison of clinical outcomes between blind and non-blind trial for all randomised women was performed (the intention-to-treat analysis for the effect of blinding), using the Cox proportional hazards modelling. Clinical outcomes were then compared between blind and non-blind trial separately for recruited and non-recruited women. For recruited women, separate comparisons were done for HT and non-HT arms, as well as the combined analysis, stratified by HT assignment. Interaction for blinding and treatment among recruited women was tested for, but not included in the model. In addition, comparison of outcomes between recruited and non-recruited women in the placebo and non-treatment arms was carried out.

For each of the comparison, both crude and adjusted hazard ratios with 95% confidence intervals were obtained, using Cox proportional hazards modelling.

The software used for analyses was R for Windows, version 2.8.1 [21].

## Results

### Effect of blinding on external validity of clinical outcomes

From the 2087 women randomised to the non-blind trial, and from the 2083 women randomised to the blind trial, 1001 (47.9%) were recruited to the non-blind and 777 (37.3%) to the blind trial arms (p < 0.0001 for the difference). From the 2392 women not recruited to the trial, eight women had deceased before the follow-up period (one in blind HT arm, two in non-blind HT arm, two in placebo arm and three in non-treatment arm), and two women not recruited to the placebo arm had lost health insurance (Figure 1).

The mean follow-up time from January 1, 2001 to the earliest of December 31, 2007 or loss of follow-up (death or leaving the country), was 6.89 years (minimal 0.11 years, maximal 7.0 years). When December 31, 2004 was used as the last follow-up date, the mean follow-up time was 3.97 years (minimal 0.11 years, maximal 4.0 years).

From the background characteristics recorded in the recruitment questionnaire, age, education, living place (clinic of recruitment), smoking status and time since menopause appeared to influence the probability of being recruited. Younger women were more eager to join the trial. University education increased considerably the recruitment probability in non-blind trial, with possibly only a weak effect in blind trial. Current smokers were less interested to join the non-blind trial, there was no significant difference in that respect in the blind trial. The university clinic appeared to be more successful in recruiting women to blind trial than the other clinics, with no difference found for non-blind trial (Table 1).

**Table 1:** Proportion of women with different background characteristics among recruited and non-recruited women in blind and non-blind trial arms, EPHT Trial

Background characteristics	Blind (n = 2083)			Non-blind (n = 2087)		
	
	Recruited (n = 777)	Non- recruited (n = 1306)	p-value	Recruited (n = 1001)	Non-recruited (n = 1086)	p-value
Age > 64 yrs	5.5%	9.3%	0.002	6.8%	10.1%	0.008

University education	31.8%	29.5%	0.28	33.5%	25.1%	< 0.0001

Recruited in the university clinic (living in Tartu or Tartu county)	33.3%	27.2%	0.003	29.8%	29.2%	0.77

Current smoker	15.2%	16.7%	0.39	14.7%	20.1%	0.002

> 10 yrs since menopause	28.6%	32.7%	0.06	27.9%	34.7%	0.001

At the end of the first trial year, participants were asked in a questionnaire what was their guess about the treatment they were receiving. In the blind HT arm, 48% of participants remained unclear about their treatment allocation, and 49% in the placebo arm. The proportion of women supposing to receive active treatment was 35% in the blind HT arm and 19% in the placebo arm. The number of women supposing to receive placebo was 13% in the blind HT arm and 28% in the placebo arm. About 4% of participants in both arms of the blind trial stated to be indifferent regarding the treatment allocation (Table 2). The reasons for the guesses were not queried.

**Table 2:** Participants' guess on treatment allocation (number and proportion of women) in different trial arms at the end of the first trial year, the EPHT Trial

	Don't know	Correct guess	Incorrect guess	Don't care
Blind HT	414	300	117	36
N = 867	48%	35%	13%	4%

Blind placebo	375	213	144	33
N = 765	49%	28%	19%	4%

Adherence rate was similar in blind and non-blind HT arms, but lower in the placebo arm. However, the use of placebo prevented contamination, because more women in the non-treatment arm than in the placebo arm started using prescribed HT [15]. The proportion of adherent women in the HT arms throughout the whole trial was 30%, and 23% in the placebo arm. In the non-treatment arm, 8% of women started using prescribed HT, and 4% in the placebo arm. Adherence and contamination rates in different arms throughout the trial have been reported earlier [15].

The intention-to-treat analysis among all randomised women did not show differences between hazard rates for any outcome event between women randomised to blind and non-blind trial (crude HR between blind and non-blind trial for coronary heart disease was 0.91 (95% CI: 0.81-1.02), for cancer 0.93 (95% CI: 0.73-1.20), for cerebrovascular disease 0.97 (95% CI: 0.80-1.18), for bone fractures 1.07 (95% CI: 0.93-1.23), for total mortality 1.10 (95% CI: 0.78-1.54), and for all outcomes combined 0.98 (95% CI: 0.90-1.07). The results were similar in the adjusted analysis and in separate analyses for HT and non-HT arms.

**Figure 2:**
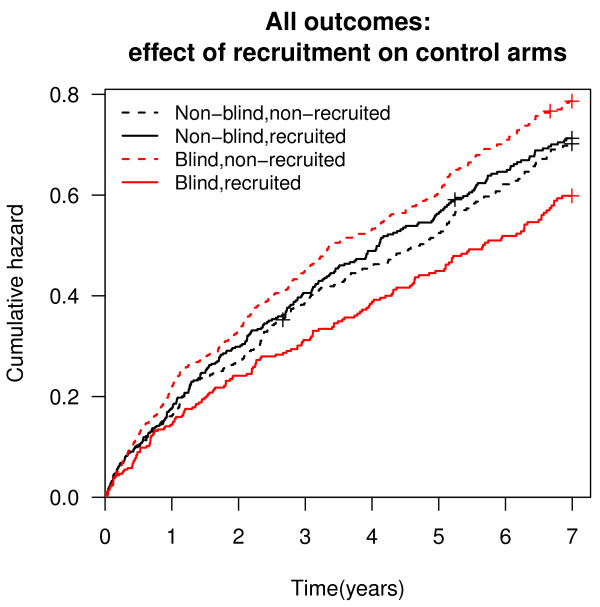
**All outcomes: effect of recruitment on control arms**.

**Figure 3:**
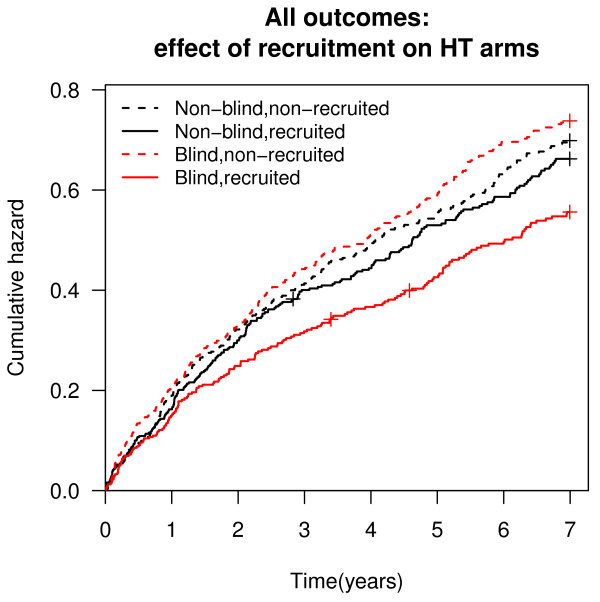
**All outcomes: effect of recruitment on HT arms**.

The difference for all outcomes combined between the women recruited and those not recruited was bigger in the blind trial than in non-blind trial, both in treatment and non-treatment arms (Figures 2, 3).

The comparison of different outcome events among recruited and non-recruited women in the placebo arm showed a difference as regards to cerebrovascular disease events (HR 0.43; 95% CI: 0.26-0.71) and all outcomes combined (HR 0.76; 95% CI: 0.63-0.91). There was no difference in outcome events among women recruited or not recruited to the non-treatment arm for any of the outcomes studied (Table 3). The sensitivity analysis of the stroke and cerebral infarction events only (ICD-10 codes I60-I64) with the follow-up until 2007 showed a difference among recruited and non-recruited women both in the placebo arm (HR 0.19; 95% CI: 0.04-0.83) and non-treatment arm (HR 0.43; 95% CI: 0.20-0.93). The risk of stroke and cerebral infarction only did not differ between women who were recruited to the blind and non-blind trial (HR 0.51; 95% CI: 0.22-1.15).

**Table 3:** Crude and adjusted hazard ratios for different outcome events among recruited versus non-recruited women in placebo and non-treatment arms, EPHT Trial, 2001 to 2007

Outcome	Placebo arm		Non-treatment arm	
	
	Recruited vs non-recruited		Recruited vs non-recruited	
	
	HR (95% CI), crude	**HR (95% CI)**, adjusted*	HR (95% CI), crude	HR (95% CI), adjusted*
Coronary heart disease	0.81 (0.62-1.04)	0.80 (0.62-1.03)	1.02 (0.82-1.28)	1.06 (0.84-1.33)

Cancer	1.00 (0.58-1.70)	0.97 (0.57-1.66)	0.71 (0.42-1.20)	0.75 (0.44-1.29)

Cerebrovascular disease	0.43 (0.26-0.71)	0.44 (0.27-0.73)	0.86 (0.59-1.26)	0.90 (0.61-1.31)

Bone fractures	0.89 (0.67-1.18)	0.88 (0.67-1.17)	1.28 (0.97-1.69)	1.30 (0.98-1.72)

Total mortality	0.51 (0.22-1.19)	0.51 (0.23-1.22)	0.72 (0.33-1.55)	0.80 (0.34-1.74)

All outcomes combined	0.76 (0.63-0.91)	0.75 (0.63-0.91)	1.05 (0.89-1.25)	1.09 (0.92-1.30)

*adjusted for age, living-place, education, smoking status, and time since menopause

### Effect of blinding on internal validity of clinical outcomes

Among all the 1778 women finally recruited to the trial and with the follow-up until 2007, there was a smaller risk of coronary heart disease events (HR 0.77; 95% CI: 0.64-0.93), cerebrovascular disease events (HR 0.66; 95% CI: 0.47-0.92) and all outcomes combined (HR 0.82; 95% CI: 0.72-0.94) in the blind trial than in the non-blind trial. The combined hazard ratio for total mortality between women recruited to the blind and those recruited to the non-blind trial arms did not differ (HR 1.03; 95% CI: 0.53-1.98), with seven deaths in the placebo arm, eleven in the non-treatment arm, nine in the blind HT, and nine in the non-blind HT arm (Table 4).

**Table 4:** Crude hazard ratios for different outcome events among recruited women in blind versus non-blind trial arms, EPHT Trial: data presented for the periods from 2001 to 2004 and from 2001 to 2007

Outcome/ Time period	Recruited women			Non-recruited women
	
	Blind vs **non-blind HT**, HR (95% CI)	Placebo vs **non-treatment**, HR (95% CI)	Blind vs **non-blind trial**, HR (95% CI)*	Blind vs non- **blind trial**, HR (95% CI)
Coronary heart disease				
2001-2004	0.89 (0.66-1.19)	0.75 (0.55-1.02)	0.82 (0.66-1.01)	1.06 (0.89-1.26)
2001-2007	0.77 (0.60-1.00)	0.78 (0.60-1.01)	0.77 (0.64-0.93)	1.01 (0.87-1.18)

Cancer				
2001-2004	0.59 (0.29-1.23)	0.47 (0.21-1.04)	0.53 (0.31-0.91)	0.94 (0.66-1.33)
2001-2007	0.77 (0.44-1.33)	1.23 (0.68-2.23)	0.95 (0.64-1.42)	0.90 (0.65-1.24)

Cerebrovascular disease				
2001-2004	0.80 (0.48-1.33)	0.34 (0.18-0.67)	0.57 (0.38-0.84)	1.24 (0.92-1.68)
2001-2007	0.80 (0.52-1.24)	0.51 (0.30-0.86)	0.66 (0.47-0.92)	1.15 (0.90-1.46)

Bone fractures				
2001-2004	0.69 (0.43-1.10)	0.94 (0.65-1.37)	0.83 (0.62-1.11)	1.10 (0.87-1.38)
2001-2007	0.96 (0.69-1.35)	0.90 (0.67-1.21)	0.93 (0.74-1.16)	1.17 (0.97-1.42)

Total mortality				
2001-2004	0.73 (0.18-3.07)	0.27 (0.03-2.32)	0.51 (0.16-1.63)	0.90 (0.52-1.67)
2001-2007	1.22 (0.49-3.08)	0.86 (0.33-2.22)	1.03 (0.53-1.98)	1.02 (0.69-1.52)

All outcomes combined				
2001-2004	0.81 (0.65-1.02)	0.75 (0.59-0.93)	0.78 (0.66-0.91)	1.10 (0.97-1.25)
2001-2007	0.83 (0.69-1.02)	0.81 (0.67-0.98)	0.82 (0.72-0.94)	1.08 (0.97-1.21)

*stratified by treatment

For women recruited to the placebo arm there was a smaller risk of cerebrovascular disease events (HR 0.51; 95% CI: 0.30-0.86) and for all outcomes combined (HR 0.81; 95% CI: 0.67-0.98), in comparison with the women recruited to the non-treatment arm. For women recruited to the blind HT arm, there was a smaller risk for coronary heart disease events (HR 0.77; 95% CI: 0.60-1.00) than for women recruited to the non-blind HT arm.

Among women not recruited to the trial, there were no differences between the blind or non-blind trial for any of the outcomes studied (Table 4). Adjustment for differences in background characteristics did not change the results (data not shown).

If restricting the analysis with the year 2004 when unblinding occurred after stopping trial treatment, there were less cancer cases (HR 0.53; 95% CI: 0.31-0.91), less cerebrovascular disease events (HR 0.57; 95% CI: 0.38-0.84) and fewer outcomes combined (HR 0.78; 95% CI: 0.66-0.91) among women recruited to the blind trial in comparison with those recruited to the non-blind trial. Among women recruited to the placebo arm, there were less cerebrovascular disease events (HR 0.34; 95% CI: 0.18-0.67) and all outcomes combined (HR 0.75; 95% CI: 0.59-0.93) until the end of 2004 than among women in the non-treatment arm (Table 4).

### Effect of blinding on subjective outcomes, and use of health services

For subjective outcomes, the detailed results have been published elsewhere: as regards symptom reporting and quality of life, there were no differences between the blind and the non-blind HT arms or placebo and non-treatment arms [13]. The use of health services did not differ between the blind HT arm and the placebo arm, but the number of health care visits was much higher in the non-blind HT arm than in the non-treatment arm [12].

## Discussion

### Summary of the key findings

As far as we know, there are no earlier reports on the impact of blinding on trial outcomes. The experience from the EPHT Trial showed a difference between the blind and non-blind trial in the number of coronary heart disease events, cerebrovascular disease events and all outcomes combined among recruited women, but not for cancer, bone fractures and total mortality, indicating that blinding may influence validity of results, and the effect may vary for different outcomes.

### Strengths and limitations of the study

Randomisation of eligible participants was unconventionally carried out before joining the trial in order to study the impact of blinding on recruitment. This resulted in losses among randomised participants. Probability of being recruited was found to be dependent on several background characteristics with the effect being different for women randomised to blind or non-blind trial. This may have caused a different selection bias in the blind and non-blind trial as compared to the target population, grounded on a preference effect [22]. From the background characteristics studied, the participant's education, smoking status and the trial setting (living in the university clinic area) influenced the probability to be recruited and might have thus influenced the external validity of outcomes due to blinding. Higher recruitment rates to the blind trial in the university clinic may indicate that trials carried out in university clinics recruit different participants than trials in other clinics.

The comparison between recruited and non-recruited women in the placebo arm indicated a smaller risk among recruited women for cerebrovascular diseases and all outcomes combined. In the non-treatment arm, no difference between recruited and non-recruited women was observed. This suggests that the differences between blind and non-blind trial can be explained by differential post-randomisation selection: the women who joined the blind trial may have had smaller baseline risks for outcome events than those who joined the non blind trial. In this case, comparison of the risks of outcome events among non-recruited women who were assigned to blind and non- blind trial should result in hazard ratios that are approximately inverse of the corresponding hazard ratios in recruited women. Here, however, this is only partly the case, as for coronary heart disease and cerebrovascular disease the hazard ratios for non-recruited women are not significant and the point estimates are considerably smaller than the inverse of the corresponding hazard ratios in recruited women. This supports the hypothesis that the differences among recruited women in blind and non-blind trial cannot be entirely explained by differential selection, and that blinding may influence also internal validity.

Analysis of outcome events among all randomised women did not show any differences between trial arms. So there is no clear evidence of a "causal effect" of blinding - the risk of outcome events for any particular women being altered by random assignment to either blind or non blind trial. However, for coronary heart disease the difference is of borderline significance and confidence intervals for other outcome events are very wide - so we cannot entirely exclude the possibility of a causal effect of blinding.

Among recruited women, there were less cerebrovascular disease events and all outcomes combined in the placebo arm than in the non-treatment arm, and less coronary heart disease events in the blind than in the non-blind HT arm. A separate analysis with a shorter follow-up period until trial participants and physicians became unblinded showed less cancer cases, cerebrovascular disease events and all outcomes combined in blind trial. The differences in the outcomes between the shorter and longer follow-up period support the hypothesis that in addition to selection bias, there is a potential reporting and/or observer bias in blinded studies. For cancer, blinding may have caused a delay in diagnosis; for cerebrovascular disease, the additional sensitivity analysis for only 'hard' outcomes like stroke and brain infarction did not show a difference between blind and non-blind trial among recruited women.

We also note that the difference in clinical outcomes between the placebo and non-treatment arm was inconsistent for various outcomes which were studied. These differences pinpoint the importance of the behavioural component, the attitudes of physicians and patients, sometimes called the placebo effect [23]. In the Women's Health Initiative (WHI) trial, where 44.4% of women on active treatment were unblinded (versus 6.8% on placebo), [24] detection bias was estimated to have caused higher detection rates of otherwise unrecognised acute myocardial infarction among HT users [25].

The results of the WHI trial were not discussed widely in Estonia, and the preterm stopping of that trial did not receive much media coverage, if any. All trial participants received a thorough medical check-up at the closure visit. Therefore, we presume that the preterm stopping of the trial did not influence further care-seeking by trial participants.

## Conclusions

### Implications in the context of the evidence

The clinical usefulness of the results from clinical trials depends on their external and internal validity. In everyday practice, patients and physicians are not blinded. In contrast with the earlier studies, the experience from the EPHT trial did not show an exaggerated treatment effect within the non-blind trial in comparison with the blind trial [26,27]. Patients have preferences that are related to the design of a clinical trial. However, the trial design is also related to its purpose. Blind settings are preferable to test the efficacy of a new treatment whereas the effectiveness of treatment and the use of health care resources may be more properly studied within non-blind settings [28]. Our study demonstrates that non-blind randomised trials may be suitable for postmarketing trials or health services research in general. Meta-analyses incorporating data both from clinical trials and observational studies should take into account the possible difference in the outcomes not only due to randomisation, but also due to blinding.

### Unanswered questions and future research directions

In summary, our findings suggest that the participants as well as the outcomes in the blind trials may be different than those in non-blind trials, even if the target population is the same [29]. The effect of blinding may vary for different outcome events. How much of this results from selection bias for women with different background characteristics, from difference in seeking medical advice due to health symptoms because of knowing the treatment or not, or from a delay in diagnoses due to the effect of blinding on the attitudes of physicians and patients needs further research.

## Competing interests

The authors declare that they have no competing interests.

## Authors' contributions

PV was responsible for the trial coordination, drafting the manuscript and contributed to the data analysis. KF was responsible for the analysis of data. EH and MH were responsible for the study design. EH, KF and MH contributed to drafting the manuscript and interpretation of results. PV is the guarantor for the study. All authors read and approved the final manuscript.

## Pre-publication history

The pre-publication history for this paper can be accessed here:

http://www.biomedcentral.com/1471-2288/12/44/prepub
